# Event and Comment

**Published:** 1907-10-15

**Authors:** 


THE
### DENTAL REGISTER.

Vol. LXI. October 15, 1907. No. 10.

### EVENT AND COMMENT.

### Oral Hygiene for School Children.

We print in this issue two good papers on the subject of doing some organized work looking toward dental hygiene in the public schools. This question comes up once in a while for discussion in our dental societies, but for some reason nothing seems to be gained by the discussion or at least no considerable progress so far as practical application of the theories projected or resolutions adopted are concerned. There seems to have been a more general interest in the subject during the past year than ever before. In Germany, France and England, hospitals for the treatment of the teeth of poor school children in the public schools have been organized under government patronage or by private endowment. It is somewhat curious that in this country where dentistry has taken such high ground that no considerable effort has been made to start similar hospitals. A few private hospitals have been organized, and during a brief period have accomplished good work in serving poor school children, but they do not endure. Consequently we are not able to decide whether the dental hospital will ever take the place of medical hospitals in the treatment of diseases of the mouth and teeth. In the cities where are located dental colleges some work has been done for the poor of all ages and nationalities, but no school has undertaken to care for all the inmates of the eleemosynary institutions in its territory, much less has there been a concerted effort to look after the children in the public schools.

It may be that the task is too large to be handled by the students, or that the colleges cannot afford to take up this work seriously because of the expense involved in supplying material and men to adequately care for it.

There is no question but the poor children need this professional care badly, and it is seriously to be regretted that no way seems open for providing for the expense involved. We trust the time may not be far distant when an enlightened public will see that it is of great importance to take care of the poor children's teeth at public expense. Many of these children when grown will become useful citizens and they should be provided with a body in good health that shall enable them to take care of themselves.

But an equally important feature of this campaign is educational and is suggested for chilrden in the public schools, whose parents are either negligent of their childrens best interests or do not know that anything can be done to stop the ravages of decay in the teeth. There seems to be a lack of attention to the care of children's teeth even in intelligent families that can hardly be reconciled with efforts made to do for them other things of less importance. We can't help concluding that parents are not purposely negligent, but have not yet reached that high plane of civilization which goes with the tooth brush. It seems like an enormous undertaking to start out to educate every child in the principles of oral hygiene, but we shall never accomplish much unless some better effort is put forth in this direction than has yet been done. Dentists of to-day know what ought to be done and it is their duty to force on the public that knowledge which they possess and which makes for the public's good. There is a foolish notion among school boards that dentists are seeking their personal ends in trying to do any kind of educational work in the public schools. This must be overcome by a concerted movement by dentists in every town and city backed up by local and state organizations of every kind. If we can get the children to take care of their teeth we shall have made an enormous gain for good health.

